# Candidemia due to *Candida guilliermondii* in an immuno-compromised infant: a case report and review of literature

**DOI:** 10.18502/cmm.5.1.535

**Published:** 2019-03

**Authors:** Fatemeh Ahangarkani, Hamid Badali, Mohammad Sadegh Rezai, Tahereh Shokohi, Zahra Abtahian, Hassan Mahmoodi Nesheli, Hossein Karami, Emmanuel Roilides, Ahmad Tamaddoni

**Affiliations:** 1 Student Research Committee, Mazandaran University of Medical Sciences, Sari, Iran; 2 Invasive Fungi Research Center/Department of Medical Mycology, School of Medicine, Mazandaran University of Medical Sciences, Sari, Iran; 3 Pediatric Infectious Diseases Research Center, Mazandaran University of Medical Sciences, Sari, Iran; 4 Infectious Diseases and Tropical Medicine Research Center, Shahid Beheshti University of Medical Sciences, Tehran, Iran; 5 Amirkola Children Hospital, Babol University of Medical Sciences, Babol, Iran; 6 Thalassemia Research Center, Hemoglobinopathy Institute, Mazandaran University of Medical Sciences, Sari, Iran; 7 3rd Department of Pediatrics, Infectious Diseases Section, Faculty of Medicine, Aristotle University School of Health Sciences, Thessaloniki, Greece

**Keywords:** Candida guilliermondii, Candidemia, Cancer, Pediatric

## Abstract

**Background and Purpose:**

Candidemia is a life-threatening fungal infection with significant mortality and morbidity in neutropenic individuals, immunosuppressive chemotherapy recipients, and broad-spectrum antibiotics consumers. The epidemiology and antifungal susceptibility testing of non-*albicans Candida* species have been poorly studied. These species are characterized by low susceptibility to azoles and echinocandins. Herein, we report the first pediatric case of candidemia due to *C. guilliermondii* in Iran and review the literature on fungemia caused by *C. guilliermondii*.

**Case report:**

We presented the first candidemia case due to *C. guilliermondii *in a 4-month-old male infant with neuroblastoma in Iran. This study also involves a comprehensive literature review on fungemia caused by *C.* *guilliermondii *during a period of 18 years (i.e., 2000-2018) to discuss the epidemiology, clinical features, and treatment of this disease. The literature review resulted in the identification of 501 cases of candidemia caused by *C. guilliermondii*. Most of the patients were adults and had multiple risk factors. However, the main risk factors were significantly related to cancer chemotherapy, followed by central venous catheter use and Intensive Care Unit admission. Mortality rate due to this disease had a range of 3.4-66.6%, in this regard, the patients with cancer had the highest mortality rate.

**Conclusion:**

Given the high mortality of candidemia, the early diagnosis of this infection and timely initiation of antifungal therapy significantly improve the patients’ survival rate and result in better outcomes. Consequently, it is highly recommended to monitor the local epidemiology of this life-threatening infection and raise awareness in this regard.

## Introduction

Candidemia is a life-threatening fungal infection with significant morbidity and mortality among pediatric patients, especially among those subjected to intravenous catheters for a long time, hematopoietic stem cell transplantation, and immunosuppressive therapy or the patients with severe immunodeficiency and cancer [1]. Although *Candida albicans* is generally the most frequent cause of candidemia, non-*albicans* *Candida* species (i.e., *C. glabrata*, *C. tropicalis*, *C. krusei*, *C. parapsilosis*, *C. auris,* and *C. guilliermondii*) have become more frequent and have been recognized as emerging pathogens in cancer patients [1, 2]. 

Accordingly, the incidence rate of candidemia due to *C. guilliermondii* ranges from 0.6% in North America to 3.7% in Latin America. In addition, the decreased susceptibility of this pathogenic yeast to fluconazole has been observed in different geographical areas [1-4]. However, the epidemiology of candidemia due to *C. guilliermondii* has been underestimated so far. Herein, we report the first pediatric case of candidemia due to *C.* *guilliermondii *in Iran and present a comprehensive literature review regarding fungemia caused by C. *guilliermondii*.

## Case report

Our case was a 4-month-old male infant with neuroblastoma undergoing chemotherapy referred to the Oncology Department of Amirkola Children’s Hospital, Mazandaran, Iran, with fever and neutropenia, without any obvious source of infection. The patient had undergone surgery for neuroblastoma 2 months prior. Laboratory examinations showed the C-reactive protein level of 76 mg/L, white blood cell count of 1.8×10^3^/ μl (i.e., leukopenia), neutrophil count of <500 cell/μl, hemoglobin level of 6.5 g/dl, and platelet count of 134×10^3^/ μl. The blood samples were collected aseptically by arterial puncture in BD BACTEC Plus Aerobic/F culture bottles (Becton Dickinson and Company Spark, MD 21152, Shannon, County Clare, Ireland) and incubated in a BACTEC culture system (Becton Dickinson Microbiology Systems).

The patient was prescribed ciprofloxacin prophylaxis due to mucositis; in addition, empirical therapy with ceftazidime and vancomycin was instituted for up to 7 days; however, his condition deteriorated rapidly. Initial blood cultures were negative for bacteria, whereas two consecutive blood cultures were positive for yeast-like fungi. Positive blood cultures were subcultured on CHROMagar *Candida* (bioMe´rieux) and resulted in the emergence of smooth colonies with white to cream colors after 24 h in dark. *Candida* species were initially identified based on conventional assays.

Voucher strains were deposited into the reference culture collection under the accession number IFRC2085. In addition, identification at the species level was performed by using DNA sequencing. Genomic DNA was extracted from 2 to 3-day-old Sabouraud dextrose agar cultures with an UltraClean Microbial DNA Isolation Kit (Mo Bio Laboratories) according to the manufacturer’s protocol, and then stored at -20°C prior to use. The internal transcribed spacer (ITS) was amplified and sequenced using primers ITS5 and ITS4 as previously described [5].

Briefly, the amplification of ITS rDNA was performed using a cycle of 5 min at 94°C for primary denaturation, followed by 40 cycles at 94°C for 30 sec, 52°C for 30 sec, and 72°C for 80 sec and a final 7-min extension step at 72°C. The sequence data were adjusted using Lasergene SeqMan software (version 9.0.4, DNASTAR) and compared with the data of GenBank through local BLAST with a molecular database maintained for research purposes at the CBS-KNAW Fungal Biodiversity Centre, Utrecht, Netherlands. The DNA sequence of the ITS rDNA region matched that of *C. guilliermondii* (MH714912) by showing 99.9% similarity with the ex-type strain. 

In vitro antifungal susceptibility test was also performed according to the documents M27-A3 and M27-S4 of the Clinical and Laboratories Standards Institute. For the preparation of the microdilution trays, amphotericin B (Sigma, St. Louis, MO, USA), fluconazole (Pfizer, Groton, CT, USA), itraconazole (Janssen research foundation, Beerse, Belgium), voriconazole (Pfizer), and caspofungin (Merck, Whitehouse Station, NJ, USA) were obtained from their respective manufacturers as reagent-grade powders. The minimum inhibitory concentrations for amphotericin B, fluconazole, itraconazole, voriconazole, and caspofungin were obtained as 0.063, 4, 2, 0.25, and 0.5 µg/ml, respectively.

The patient was empirically treated with 0.75 mg/kg/day amphotericin B deoxycholate intravenously, which is a regimen frequently used as standard therapy for candidaemia in Iran. After treatment with amphotericin B for a week, two sequential blood cultures remained negative. The patient was successfully treated and showed no relapse during the two-week follow-up. This report was approved by the Ethics Committee of Mazandaran University of Medical Sciences, Mazandaran, Iran. In line with the principles of research ethics, written informed consent was obtained from the parents of the patient.

## Discussion

*Candida guilliermondii* complex comprising several species, namely* C. guilliermondii*, *C.* *fermentati*, *C. carpophila*, and *C. xestobii,* is an uncommon, newly emerging, and rare agent of candidemia, with low incidence (1-3%), especially in immunocompromised hosts, transplant recipients, and critically ill patients [3]. 

Limited cases of invasive candidiasis caused by *C. guilliermondii *complex have been reported in the past because of its low pathogenicity. However, recently, there is an increasing number of reports regarding the bloodstream infections due to this complex [4]. In addition, due to resistance or decreased susceptibility to antifungal agents, *C. guilliermondii* complex has been proposed to be a re-emerging pathogen in high-risk patients. 

Table 1 summarizes all reported cases of candidemia due to *C. guilliermondii* in English literature with the patients’ demographic characteristics (e.g., age, gender, source, and location) and clinical data (e.g., underlying condition, risk factors, and outcomes). Most of these patients were adults and had multiple risk factors. The main risk factors were significantly related to cancer patients undergoing chemotherapy, followed by central venous catheter users and ICU patients (Table 1). In the reviewed articles, the mortality rate had a range of 3.4-66.6%. In this regard, this infection had the mortality rates of 11.76-66.6%, 13.6-54%, 16.66-18.8%, 59.25%, and 3.4% in Japan, Spain, Taiwan, United States, and Italy, respectively (Table 1). 

Cancer patients suffering from this infection had a high rate of mortality. While the majority of *C. guilliermondii* fungemia cases have been described in adults with cancer, few cases have been published in pediatric patients. Peman et al. reported seven cases of *C. guilliermondii* fungemia during a 12-year period, five cases of which occurred in children [3]. In contrast, in a meta-analysis on the epidemiology of candidemia in Iran, *C. guilliermondii* accounted for 2 (3.8%) cases of infection in adults [6]. 

**Table 1:** Cases of candidemia caused by *Candida guilliermondii* reported in the literature

**Number**	**Year of evaluation**	**Country**	**Underlying condition and predisposing factors**	**Pediatric/ adult**	**Number ** ^1^ **/total**	**Resistant to azoles**	**Resistant to echinocandins**	**Mortality** **rate**	**Reference**
	2018	Iran	- Cancer - Chemotherapy - Surgery - Neutropenia	4-month-old infant	1	0	0	-	Current case
	2007-2016	Japan	- Hematological disorder - Surgery - ICU exposure	Adult	17/121	17.6-13.3%	0	11.76%	[1]
	2008-2014^2^	Japan	- Hematopoietic stem cell transplant recipients	Adult	3/22	NS	NS	66.6%	[8]
	2006-2015	Turkey	- Cancer - TPN - CVC - ICU exposure - Chemotherapy	Both	141/NS	26.08%^3^	NS	NS	[2]
	2007-2014	Spain	- Cancer - Immunosuppressive therapy - Neutropenia - Chemotherapy	Both	22/NS	72%	0	13.6%	[4]
	2005-2014	USA	- Cancer - ICU exposure - Exposure to steroids	Pediatric	3/192	NS	NS	0	[9]
	2006-2012	Italy	- Cancer	Pediatric	1/28	NS	NS	NS	[10]
	2003-2015	Taiwan	- Cancer - CVC	Both	36^4^	0	4.5-22.7%	16.66%	[11]
	2007-2014	Taiwan	- Cancer	Adult	11/21	81%	36%	18.18	[12]
	1998-2013	USA	- Cancer - Neutropenia - TPN - Steroid exposure	Adult	28/79^5^	17-24%	3.7%	59.25%	[7]
	2002-2007	Brazil	- Hematological disorder	Both	6/67	0	NS	NS	[13]
	March-April 2012	Spain	- TPN - Steroid exposure - CVC - Surgery - Broad-spectrum antibiotic exposure	Adult	4/13	0	0	54%	[14]
	2007-2013	Japan	- Cancer - Chemotherapy - CVC - TPN - Neutropenia - Immunosuppressive therapy - ICU exposure	Both	16/66	12.5%	6.2%	18.75%	[15]
	2009-2012	Taiwan	- Elderly patients - Cancer - Chemotherapy	Adult	2/181	NS	NS	NS	[16]
	2009-2012	Taiwan	- Cancer - CVC - Neutropenia - Use of steroid - Recent abdominal surgery - Chemotherapy - TPN - Broad-spectrum antibiotic exposure	Adult	2/209	NS	50%	NS	[17]
	2010-2011	Spain	- NS	NS	13/781	0	0	NS	[18]
	2007-2013	Spain	- Cancer	NS	7/593	42.85%	0	NS	[19]
	2009-2012	India	- Patients with injuries	NS	4/212	NS	NS	NS	[20]
	2007-2010	Brazil	- NS	Pediatric	5/104	NS	NS	NS	[21]
	2004-2008	USA	- Cancer - Stem cell transplantation - Chemotherapy	Both	9/2496	0	0	NS	[22]
	2009-2011	China	- Cancer - CVC - Preterm infants with low birth weight	Both	39/238	NS	NS	NS	[23]
	2009-2010	France	- Immunosuppressive drugs user - CVC	Both	1/189	NS	NS	NS	[24]
	2004-2006	Taiwan	- NS	Both	6/152	NS	0	NS	[25]
	2006-2007	Brazil	- Cancer	Pediatric	9/20	NS	NS	NS	[26]
	1995 to 2006	Spain	- Cancer - CVC - Chemotherapy - ICU stay	Both	7/NS	42.85%	0	28.57%	[3]
	2003-2004	Brazil	- NS	Pediatric	64/149	NS	NS	NS	[27]
	2001-2006	Ireland	- NS	Both	4/151	25-75%	NS	NS	[28]
**Table 1. **Continued.									
	1983 -2005	Italy	- Cancer - Chemotherapy	NS	29/243	NS	66%	3.4%	[29]
	1998-2004	Brazil	- Cancer - Prior use of antibiotics - CVC - Use of steroid - Chemotherapy	Both	1/131	0	NS	NS	[30]

1 Number of *C. guilliermondii* episodes;

2 Breakthrough candidemia was evaluated in this study;

3 AFST performed for 46 isolated in this study;

4 From January 2003 to September 2015, 4213 episodes of candidemia were identified, 1.9% (79/4213) of which were due to *C. guilliermondii* (however, only 36 cases were characterized and enrolled in the present study);

5 In this study, only candidemia caused by uncommon *Candida* species were evaluated; ICU: intensive care unit, NS: not specified, TPN: total parenteral nutrition, CVC: central venous catheter

Our patient was an infant and had a history of chemotherapy and surgery. The epidemiology and antifungal susceptibility testing of *C. guilliermondii* complex have been poorly studied. This complex is characterized by low susceptibility to azoles and echinocandins. In line with our study demonstrating the susceptibility of *C. guilliermondii* to amphotericin B and its resistance to fluconazole, numerous studies have demonstrated high MICs for azoles [3-10]. Our literature review showed that the rates of high MICs for azoles and echinocandins were 0-81% and 0-50%, respectively. Although echinocandins therapy is highly effective, emerging drug resistance is a growing threat to successful clinical management. 

## Conclusion

This is the first report describing candidemia due to *C. guilliermondii* in a pediatric patient in Iran. Given the high mortality rate of this infection, the early diagnosis and initiation of appropriate antifungal therapy for this infection significantly improve the patients’ survival rate and result in better outcomes. It is highly recommended to monitor the local epidemiology of this life-threatening infection and obtain awareness in this regard.
